# p19^ Arf^ Suppresses Growth, Progression, and Metastasis of Hras-Driven Carcinomas through p53-Dependent and -Independent Pathways

**DOI:** 10.1371/journal.pbio.0020242

**Published:** 2004-08-17

**Authors:** Karen S Kelly-Spratt, Kay E Gurley, Yutaka Yasui, Christopher J Kemp

**Affiliations:** **1**Fred Hutchinson Cancer Research CenterSeattle, WashingtonUnited States of America

## Abstract

Ectopic expression of oncogenes such as *Ras* induces expression of p19^Arf^, which, in turn, activates p53 and growth arrest. Here, we used a multistage model of squamous cell carcinoma development to investigate the functional interactions between Ras, p19^Arf^, and p53 during tumor progression in the mouse. Skin tumors were induced in wild-type, *p19^Arf^*-deficient, and *p53*-deficient mice using the DMBA/TPA two-step protocol. Activating mutations in *Hras* were detected in all papillomas and carcinomas examined, regardless of genotype. Relative to wild-type mice, the growth rate of papillomas was greater in *p19^Arf^-*deficient mice, and reduced in *p53*-deficient mice. Malignant conversion of papillomas to squamous cell carcinomas, as well as metastasis to lymph nodes and lungs, was markedly accelerated in both *p19 ^Arf^-* and *p53*-deficient mice. Thus, p19^Arf^ inhibits the growth rate of tumors in a p53-independent manner. Through its regulation of p53, p19^Arf^ also suppresses malignant conversion and metastasis. p53 expression was upregulated in papillomas from wild-type but not *p19^ Arf^*-null mice, and p53 mutations were more frequently seen in wild-type than in *p19^ Arf^*-null carcinomas. This indicates that selection for *p53* mutations is a direct result of signaling from the initiating oncogenic lesion, *Hras,* acting through p19^Arf^.

## Abstract 2

A squamous cell carcinoma model shows Ras mutation not only initiates tumor development but, through Arf and p53, directly influences the subsequent evolutionary trajectory of the tumors

## Introduction

Tumor development and metastasis is a multistep process of somatic cell evolution that includes uncontrolled proliferation, impaired apoptosis, loss of differentiation, immortalization, neovascularization, invasion, and metastatic spread (Hanahan and Weinberg 2000). This evolutionary transformation can be operationally divided into distinct stages, including initiation, promotion, progression, and metastasis (DiGiovanni 1992). Mutations in both oncogenes and tumor suppressor genes are found in end-stage tumors, implying their causal role in tumor development. However, the association of mutations in specific genes with specific phenotypic states during tumor progression is poorly characterized for most human solid tumors. It is also largely unknown whether each mutation is an independent event or whether there is a preferred sequence or combination of mutations that is favored. The purpose of this study is to investigate the functional interactions between the mutational activation of the oncogene *Ras,* and two tumor suppressors, *p19 ^Arf^,* and *p53,* using a multistage epithelial tumor model.

*Ras* is among the most frequently mutated oncogenes in human cancer, with approximately 30% of tumors carrying an activating mutation in one of three family members, *Hras, Kras,* or *Nras* (Bos 1989). Cancer-associated mutations in *Ras* result in constitutively active Ras protein. Ras is a nodal signaling molecule that regulates multiple signaling pathways, leading to profound changes in cellular proliferation, apoptosis, differentiation, senescence, cytoskeletal organization, adhesion, and migration (Campbell et al. 1998). Ras has also been shown to induce invasiveness and metastasis of cancer cells (Pozzatti et al. 1986; Webb et al. 1998; Varghese et al. 2002). These pleiotropic effects suggest Ras may influence multiple steps in tumor progression.

*p53* is the most frequently mutated tumor suppressor gene in human cancer, with more than 50% of tumors showing mutations (Hollstein et al. 1994). p53 is a nodal signaling protein that coordinates the cellular response to different types of stress, including oncogene activation, DNA damage, abnormal cell adhesion, altered ribonucleotide pools, hypoxia, and redox stress (Ko and Prives 1996; Giaccia and Kastan 1998). These stress stimuli are thought to activate p53 by inducing posttranslational modifications that stabilize p53 and enhance its ability to act as a transcription factor (Siognov and Haupt 1999; Vousden and Lu 2002). Loss of p53 function leads to loss of cell cycle checkpoints, impaired apoptosis, genomic instability, and tumor progression. However, a major unresolved issue is, of the many signals that have been shown to activate p53 using a variety of model systems, which one regulates p53 during autochthonous tumor progression.

A mechanistic connection between Ras signaling and activation of p53 that involves the tumor suppressor p19^Arf^ was recently established. p19^Arf^ (p14^Arf^ in humans; Stott et al. 1998) is encoded by the *p16^Ink4a^/p19^ Arf^* locus, but because it is transcribed in an alternative reading frame, the protein product is unrelated to the p16^Ink4a^ protein (Quelle et al. 1995; Kamijo et al. 1997). Deletions or mutations at the *p16^Ink4a^/p19^ Arf^* locus are frequently (more than 50% of cases) seen in human tumors (Ruas and Peters 1998). p19^Arf^ was established as a bona fide tumor suppressor in studies showing that mice lacking p19^Arf^ are highly susceptible to spontaneous tumorigenesis (Kamijo et al. 1997). In vitro studies had shown that enforced expression of oncogenes such as *Ras, c-Myc,* and *E1A* activated p53 and induced growth arrest, senescence, or apoptosis depending on the cell type or oncogene used (Lowe and Ruley 1993; Hermeking and Eick 1994; Serrano et al. 1997). These cellular responses were impaired in cells lacking p53, indicating that functional p53 was required. The involvement of p19^Arf^ was first suggested by experiments showing that enforced expression of Ras, Myc, and E1A in cells induced p19^Arf^, leading to G1 and G2 cell cycle arrest that was p53-dependent (Kamijo et al. 1997; Stott et al. 1998). Cells lacking p19^Arf^ showed impaired p53 induction in response to these oncogenes, and, like p53-deficient cells, escaped growth arrest and were immortalized (Zindy et al. 1998; de Stanchina et al. 1998; Palmero et al. 1998; Lin and Lowe 2001; Ferbeyre et al. 2002). In vivo evidence linking oncogene signaling to p19^Arf^ and p53 was obtained in a lymphoma model. B-cell lymphomas from transgenic Eμ-myc mice also show a dependence on p19^Arf^ to activate p53, and Eμ-myc mice lacking either p19^Arf^ or p53 developed lymphomas much faster (Eischen et al. 1999; Schmitt et al. 1999). p19^Arf^ regulates p53 through mutual binding to the p53 regulator Mdm2. The levels of p53 in cells are normally kept low because of feedback regulation by the Mdm2 protein (Haupt et al. 1997; Kubbutat et al. 1997). Mdm2 binds to p53 and targets it for degradation by nuclear to cytoplasmic shuttling and through the E3 ubiquitin ligase activity of Mdm2 (Roth et al. 1998; Honda and Yasuda 1999). p19^Arf^ sequesters Mdm2 from p53 and inhibits the ubiquitin ligase activity of Mdm2, resulting in increased stability and accumulation of p53 (Pomerantz et al. 1998; Tao and Levine 1999; Weber et al. 1999; Zhang and Xiong 1999).

The importance of the Ras-p19^Arf^-p53 pathway in growth arrest was established in culture systems involving the ectopic overexpression of mutant Ras in both murine embryonic fibroblasts (Ferbeyre et al. 2002; Palmero et al. 1998) and primary epidermal keratinocytes (Lin and Lowe 2001). Both in vitro and in vivo models have established that the gene dosage of mutant *Ras* is critical for its oncogenic function. A single copy of mutant *Ras* is insufficient to transform cells; at least two mutant alleles are required (Finney and Bishop 1993). Duplication or even amplification of mutant *Ras* alleles is frequently observed in tumors (Quintanilla et al. 1986; Bremner and Balmain 1990). Ras activates multiple signaling pathways, and quantitative differences in Ras activity can lead to activation of different signals and qualitatively different cellular phenotypes (Shields et al. 2000). Cell culture conditions can add additional stress signals that are known to impinge on p19^Arf^ and p53, leading to cell cycle arrest (Sherr and DePinho 2000; Lowe and Sherr 2003), and cannot recapitulate the complex cellular ecology of tumor progression. Thus, to understand the interactions between Ras, p19^Arf^, and p53 that drive tumor progression, an autochthonous tumor model is required. Since more than 90% of human cancers are epithelial in origin, a carcinoma model system is favored.

Mouse skin carcinogenesis is perhaps the best-characterized in vivo model of epithelial neoplasia and was instrumental in establishing the concepts of initiation, promotion, and progression (DiGiovanni 1992). The two-stage chemical protocol involves treatment of mice with a carcinogen, DMBA, followed by multiple applications of TPA. This treatment induces benign squamous cell papillomas, nearly 100% of which have sustained an AT mutation in codon 61 of *Hras* (Quintanilla et al. 1986). As this mutation results in constitutively active Ras protein, this protocol is ideal to study the biological consequence of Ras activation during the entire natural history of tumor progression. Papillomas consist of a series of folded epidermal or follicular hyperplasias that protrude from the skin surface. Papillomas have dysplastic characteristics including disturbed cell polarity, basal cell hyperplasia, disturbed maturational sequence, increased mitotic activity, and increased nuclear to cytoplasmic ratio (Yuspa 1994). In most strains of mice, progression of these benign papillomas to malignant squamous cell carcinomas (SCCs) is a rare and late event. SCCs are usually endophytic tumors that present as plaques with an ulcerated surface. These tumors break through the basement membrane and progressively invade the underlying dermis and subcutaneous tissues, and rarely, can metastasize to regional and distant sites. SCCs are characterized by a disorderly proliferation of epithelial cells with increased cellular atypia and abnormal mitotic figures, and are classified into four grades: well-differentiated, moderately differentiated, poorly differentiated, and spindle cell carcinoma. The unique advantage of this skin tumor model is the ability to directly observe and quantify these evolutionary stages.

Loss of p53 function is strongly associated with the benign to malignant transition of chemically induced SCCs. Mutations in p53 are seen more frequently in carcinomas than in papillomas (Burns et al. 1991; Ruggeri et al. 1991). p53 knockout mice show accelerated malignant progression of SCCs (Kemp et al. 1993). The strongest association of p53 with malignant progression was revealed in *p53^+/−^* mice, in which loss of the remaining wild-type allele of *p53* was seen in carcinomas but not papillomas, indicating a strong selective pressure to completely inactivate p53 during this transition. Accelerated malignant progression seen in the absence of p53 was accompanied by extensive loss of differentiation and lymph node metastasis, indicating that p53 inhibits multiple steps involved in malignant tumor progression.

Here, we used the mouse skin tumor model to examine the role of p19^Arf^ in regulating the levels and tumor suppressor activity of p53. In addition, we addressed the biological and functional significance of alterations in p19^Arf^, p53, or both during tumor initiation, promotion, progression, and metastasis. Similar to *p53*-null mice, loss of *p19^ Arf^* resulted in increased malignant conversion, more aggressive tumors, and frequent and rapid metastasis. However, in contrast to *p53*-null mice, *p19 ^Arf^*-null mice had greater tumor numbers and tumor growth rates, indicating additional, p53-independent tumor suppressor functions for p19^Arf^.

## Results

### Increased Papilloma Number and Size in p19^Arf^-Deficient Mice

**Figure 1:**
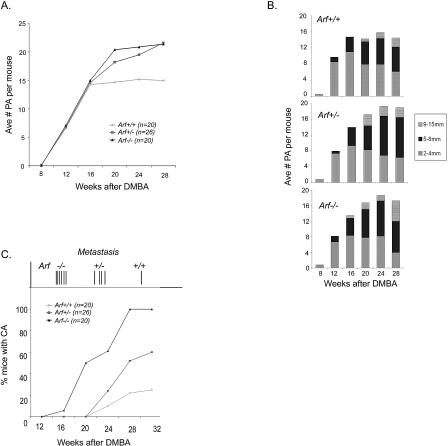
Skin Tumor Multiplicity, Size, and Progression in *p19^ Arf^*-Deficient Mice (A) Average number of papillomas (more than 2 mm in diameter) per mouse is plotted versus the number of weeks postinitiation. Both *p19^ Arf^ (Arf)^+/−^* and *p19^ Arf−/−^* mice show greater numbers of tumors than *p19^ Arf+/+^* mice. (B) Comparison of papilloma size (in mm) between *p19^ Arf+/+^, p19^ Arf+/−^,* and *p19^ Arf−/−^* mice through 28 wk postinitiation. An increase in the largest size class of tumors is seen in *p19^ Arf+/−^* and *p19^ Arf−/−^* mice but not *p19^ Arf+/+^* mice*.* (C) Percentage of mice bearing at least one carcinoma is plotted versus the number of weeks postinitiation*. p19^ Arf−/−^* mice show the shortest latency and greatest incidence of carcinoma conversion, with *p19^ Arf+/−^* mice showing an incidence between the *p19^ Arf−/−^* and *p19^ Arf+/+^* mice. Time of appearance of lymph node metastasis is noted above the graph as a vertical line for each mouse analyzed. Metastasis to lymph node occurred frequently and sooner in *p19^ Arf^*-deficient mice than in wild-type mice.

Both in vitro and in vivo studies have demonstrated that p19^Arf^ is a tumor suppressor (Eischen et al. 1999; Kamijo et al. 1999; Schmitt et al. 1999; Lin and Lowe 2001). However, other than regulation of p53, little else is known about the role of p19^Arf^ in tumor suppression. To address this, groups of *p19^ Arf+/+^*, *p19^ Arf^* ^+/−^, and *p19^ Arf−^* ^/−^ littermates were treated with a single dose of DMBA followed by twice weekly application of TPA for 15 wk (see Materials and Methods). Papillomas began to appear in all three genotypes after 9 wk of promotion. By 20 wk, *p19^ Arf+/−^* and *p19^ Arf−/−^* mice showed a significant increase in papilloma number and size compared to wild-type mice (Figure 1A). Relative to wild-type littermates, *p19^ Arf−/−^* mice had an average of 2.97 more papillomas (95% CI (0.70, 5.24); *p* = 0.010) and *p19^ Arf+/−^* mice had an average of 2.60 more papillomas (95% CI (0.05, 5.14); *p* = 0.045) in weeks 18–30 after DMBA administration. Average papilloma size was also greater in both *p19^ Arf−/−^* and *p19^ Arf+/−^* mice compared to wild-type mice (Figure 1B). This effect was seen as early as 12 wk and increased through time so that by 28 wk, 33% (47/141) of papillomas from *p19^ Arf−/−^* mice were greater than 8 mm in diameter versus 14% (38/267) from wild-type mice (*p* < 0.0001). Papillomas from *p19^ Arf^*-deficient mice measured up to 16 mm in diameter while very few papillomas on wild-type mice measured more than 9 mm. Thus, *p19^ Arf^* deficiency resulted in faster growing papillomas, indicating a role for *p19^ Arf^* in regulating the early stages of benign tumor growth.

Mutations in *Hras* are found in more than 95% of DMBA/TPA-induced skin tumors (Quintanilla et al. 1986; Kemp et al. 1993)*.* In vitro studies showed that mutant Ras induces p19^Arf^, which, in turn, inhibits Ras-induced proliferation (Sherr and Weber 2000). Thus, loss of p19^Arf^ might reduce or eliminate the need to mutate *Ras*. All papillomas from *p19^ Arf+/+^* (5/5), *p19^ Arf+/−^* (4/4), and *p19^ Arf−/−^* (5/5) mice contained the identical A→T transversion at codon 61 of *Hras,* resulting in an amino acid change from glutamine to leucine and a constitutively activated Ras protein (Quintanilla et al. 1986). Thus, mutation of *Ras* is very strongly selected for during epithelial carcinogenesis, with or without the presence of p19^Arf^, and loss of p19^Arf^ cooperates with activated Ras to accelerate tumor growth.

### Increased Malignant Progression and Metastasis in p19^Arf^-Deficient Mice

The rate of malignant conversion of papillomas to carcinomas is greatly increased in the absence of p53 function (Kemp et al. 1993). To determine if loss of p19^Arf^ had a similar effect, progression was quantified by visual inspection and confirmed by histologic analysis. The rate of conversion from papillomas to carcinomas was dramatically accelerated in p19^Arf^-deficient mice. Carcinomas developed in *p19^ Arf−/−^* mice as early as 14 wk after initiation, whereas papillomas from *p19^ Arf+/+^* mice began to convert much later, after 22 wk (Figure 1C). By 28 wk, 100% of the *p19^ Arf−/−^* mice had at least one carcinoma, compared to only 25% of the wild-type mice. The *p19^ Arf+/−^* mice showed an intermediate conversion rate, with 60% of the mice bearing at least one carcinoma, indicating an p19^Arf^ gene dosage effect on malignant progression. In addition to reducing the latency, p19^Arf^ deficiency increased the frequency of malignant conversion. The odds of developing a carcinoma within 30 wk after DMBA administration was 8.10 times higher for *p19^ Arf−/−^* mice (95% CI (1.90, 34.56); *p* = 0.005) and 3.11 times higher for *p19^ Arf+/−^* mice (95% CI (0.90, 10.77); *p* = 0.073) compared to wild-type mice. The reduced carcinoma latency and increased conversion frequency in the *p19^ Arf^*-null mice implicate loss of p19^Arf^ as a critical rate-limiting step in malignant SCC progression.

Histologic analysis revealed that the carcinomas from control mice ranged in grade from well-differentiated to poorly differentiated SCCs. Carcinomas from *p19^ Arf+/−^* and *p19^ Arf−/−^* mice also showed a range of grades but a significant number (9/12) were characterized as spindle cell carcinomas. These were characterized by packed and spindle-shaped cells with elongated pleiomorphic nuclei and abundant abnormal mitotic figures. These cells grew in a homogenous pattern with very little evidence of the cellular organization typical of low-grade tumors. These tumors showed focal areas of squamous differentiation, indicating that they were derived from squamous epithelium.

**Figure 2:**
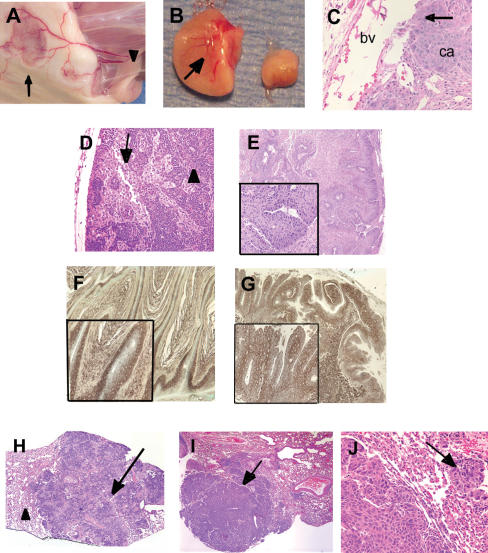
Metastasis of Primary SCC to Lymph Nodes and Lungs in *p19^ Arf^*-Deficient Mice (A) Underside of skin from tumor-bearing mouse shows newly formed blood vessels surrounding tumor site (arrow) and leading to inguinal lymph node (arrowhead). (B) Enlarged inquinal lymph node (left) containing metastatic SCC and blood vessel formation (arrow) compared to normal lymph node (right). (C) H&E stain of carcinoma section with prominent blood vessel (bv). Carcinoma cells (ca) have penetrated blood vessel wall (arrow). (D) H&E stain of lymph node bearing infiltrating SCC cells (arrow) among normal lymphocytes (arrowhead). (E) H&E stain of lymph node bearing metastatic differentiated SCC. (F) Immunostain with pan-keratin antibody of papilloma. (G) Immunostain with pan-keratin antibody of lymph node with metastatic SCC. (H and I) H&E stain of normal lung (arrowhead) with large metastatic SCC deposit (arrow). (J) H&E stain of lung metastasis with secondary site of infiltration (arrow). (D–G, J): 20× magnification. Inserts in (E–G): 40× magnification.

*p19^ Arf^* deficiency also increased dissemination and establishment of metastatic SCCs. Carcinoma-bearing p19^Arf^-deficient mice frequently presented with enlarged lymph nodes, and in several cases tumors were noted on the lungs. (Table 1; Figure 2). Histologic analysis revealed that these lymph nodes and lung tumors contained cells with features similar to the primary SCCs, including squamous differentiation, keratin pearls, high mitotic index, nuclear pleomorphism, and disturbed cell polarity (Figure 2D and 2E). Immunostaining with a keratin-specific antibody showed that these cells were epithelial in origin, confirming that they were metastatic SCCs (Figure 2F and 2G). 60% of local enlarged lymph nodes from carcinoma-bearing *p19^ Arf−/−^* mice contained such squamous carcinoma deposits, compared to 10% of those from wild-type mice (Table 1). Metastatic lesions from *p19^ Arf^*-deficient mice were seen as early as 16 wk after initiation and must have occurred very soon after or simultaneously with papilloma to carcinoma conversion (see top of Figure 1C). In contrast, only one metastatic lesion was seen in one *p19^ Arf+/+^* mice through 36 wk of observation. *p19^ Arf+^* ^/−^ mice displayed an intermediate frequency of metastasis. Newly formed blood vessels, some measuring up to 2 mm in diameter, were seen on the underside of each tumor and appeared to lead directly to the inguinal or brachial lymph node (Figure 2A and 2B). Several primary *p19^ Arf^*-deficient carcinomas showed clear evidence of penetration of tumor cells through blood vessel walls, with intravascular rafts of tumor cells seen (Figure 2C), indicating a route by which tumor cells could migrate to distant organs through the circulation. Thus, in addition to increasing benign tumor growth, loss of p19^Arf^ accelerated both benign to malignant conversion and metastatic spread of epithelial tumors. Tumors lacking p19^Arf^ have a higher potential for metastatic spread.

**Table 1:**
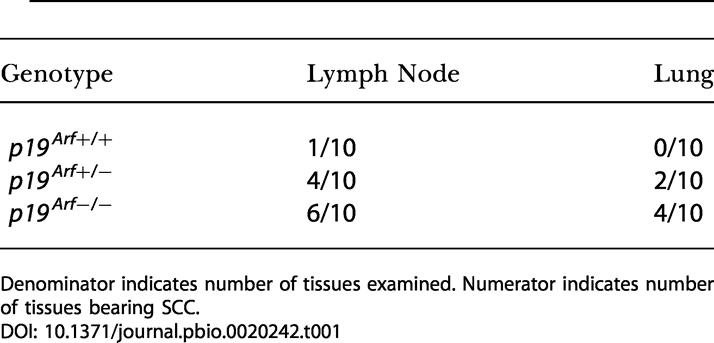
Metastatic Frequency of SCC

Denominator indicates number of tissues examined. Numerator indicates number of tissues bearing SCC

### Reduced p53 Expression in Papillomas from p19^Arf^-Deficient Mice

**Figure 3:**
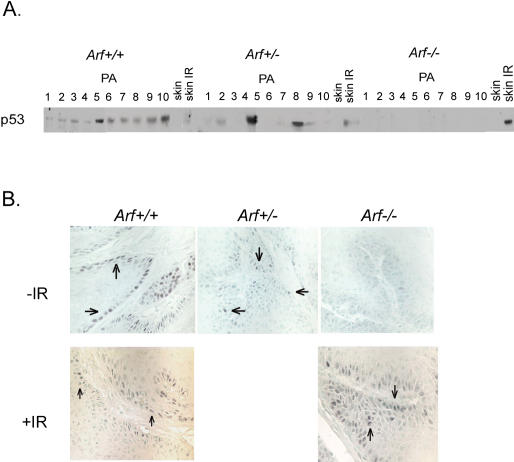
Reduced p53 Expression in Skin Tumors from *p19^Arf^*-Deficient Mice (A) Western blot analysis of nuclear lysates from skin tumors from *p19^ Arf^ (Arf)^+/+^, p19^ Arf+/−^,* and *p19^ Arf−/−^* mice using p53-specific antibody. PA, papilloma; skin IR, irradiated normal skin (B) p53 immunostain of paraffin-embedded skin tumor sections from *p19^ Arf+/+^, p19^ Arf+/−^,* and *p19^ Arf−/−^* mice (arrows indicate positive stained cells) (top). p53 immunostain of irradiated papillomas (IR) from *p19^ Arf+/+^* and *p19^ Arf−/−^* mice (bottom). p53 is not detected in normal skin or tumors from *p19^ Arf−/−^* mice, but is induced by irradiation in both normal and tumor cells from *p19^ Arf−/−^* mice.

**Figure 4:**
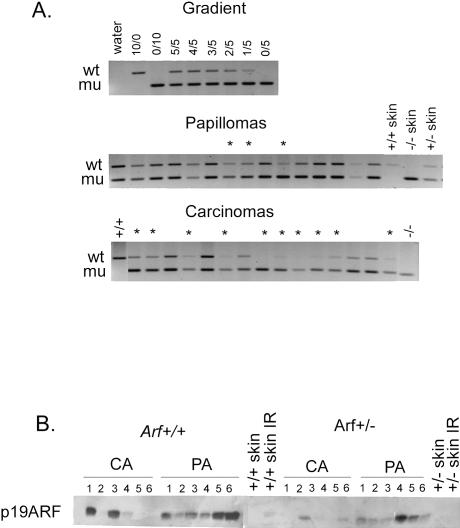
LOH of Wild-Type *p19^Arf^* Allele in *p19^Arf+/−^* Tumors (A) LOH analysis by semiquantitative PCR of the wild-type *p19^Arf^* allele in *p19^Arf+/−^* papillomas and carcinomas. Gradient made from kidney DNA used for quantitation of wt/mu ratio (top row). wt, wild-type allele; mu, knockout allele; asterisk, loss or reduction of *p19^Arf^* wild-type band. (B) Western blot analysis of nuclear lysates from papillomas (PA) and carcinomas (CA) from *p19^Arf+/+^, p19^Arf+/−^,* and *p19^Arf−/−^* mice.

The p53 expression in DMBA/TPA-induced papillomas is increased relative to adjacent normal skin (Kemp et al. 2001). As multiple signals can lead to the accumulation of p53, including activated oncogenes, DNA damage, and hypoxia, it was not clear which was operative in this setting. As nearly all carcinogen-induced papillomas carry mutations in *Hras,* we questioned whether increased p53 expression was due to signaling from Ras through p19^Arf^. Western blot analysis of nuclear lysates showed increased levels of both p19^Arf^ and p53 in wild-type papillomas compared to normal skin (Figures 3 and 4). In contrast, p53 expression was not detectable in papillomas from *p19^ Arf−^* ^/−^ mice and was intermediate and variable in papillomas from *p19^ Arf+/−^* mice. Immunostaining of paraffin-embedded sections confirmed the Western analysis, with nuclear staining of p53 detected in the epidermal cells of papillomas from wild-type mice, reduced numbers of p53-positive cells in the *p19^ Arf^* heterozygous papillomas, and undetectable p53 staining in *p19^ Arf^*-null papillomas (Figure 3B). To determine if p53 could still be induced in the absence of p19^Arf^ by an alternative pathway, tumor-bearing mice were irradiated with 4Gy ionizing radiation and sacrificed 4 h later, and their tissues were examined for p53 expression. Both Western blot analysis and immunostaining revealed prominent induction of p53 in basal cells of normal skin and papillomas from both wild-type and *p19^ Arf^*-null mice (Figure 3A and 3B). Thus, the induction of p53 seen in mutant Ras-containing tumors is due to signaling through p19^Arf^. These results provide in vivo confirmation of the model, largely derived from in vitro studies, that posits that signaling from mutant Ras acts through p19^Arf^ to induce p53. Other pathways to activate p53, such as those initiated by DNA damage, remain functional in the absence of p19^Arf^.

### Loss of the Wild-Type *p19^Arf^* Allele in Tumors from *p19^Arf+/−^* Mice Occurs During Benign to Malignant Conversion

*p19^ Arf+/−^* mice displayed an intermediate rate of papilloma to carcinoma conversion (see Figure 1C). Two genetic models could explain this heterozygous phenotype. *p19^ Arf^* could be haploinsufficient, in which case no mutation or loss of heterozygosity (LOH) should be seen in the remaining wild-type *p19^ Arf^* allele in carcinomas. Alternatively, *p19^ Arf^* could be recessive, in which case LOH or reduction to a homozygous null state would be expected. The fate of the wild-type allele of *p19^ Arf^* in tumors from heterozygous mice was assessed by semiquantitative PCR analysis of genomic DNA. Three of 15 (20%) papillomas examined showed evidence of loss of the wild-type *p19^ Arf^* allele, compared to ten of 15 (67%) carcinomas (*p* = 0.0027) (Figure 4A), indicating LOH occurs primarily during malignant conversion. We next examined p19^Arf^ expression in tumor lysates by Western blot analysis with a p19^Arf^-specific antibody. In wild-type mice, p19^Arf^ protein was elevated in all papillomas and three of six carcinomas compared to normal skin (Figure 4B). Increased expression of p19^Arf^ is consistent with activation of Ras in these tumors. p19^Arf^ expression was also increased in papillomas from *p19^ Arf+/−^* mice but not to the levels seen in wild-type mice, indicating that p19^Arf^ protein levels in tumors reflect *p19^ Arf^* gene dosage. p19^Arf^ protein was reduced or undetectable in four of six carcinomas from *p19^ Arf+/−^* mice, consistent with the LOH data. Collectively, these data indicate that p19^Arf^ expression is induced in tumors. Germline deletion of one *p19^ Arf^* allele provides a selective advantage during early tumor growth, and loss of the second allele confers an additional phenotype, destabilization of p53, and enhanced malignant progression.

### Independent Contributions of p19^Arf^ and p53 to Tumorigenesis

**Figure 5:**
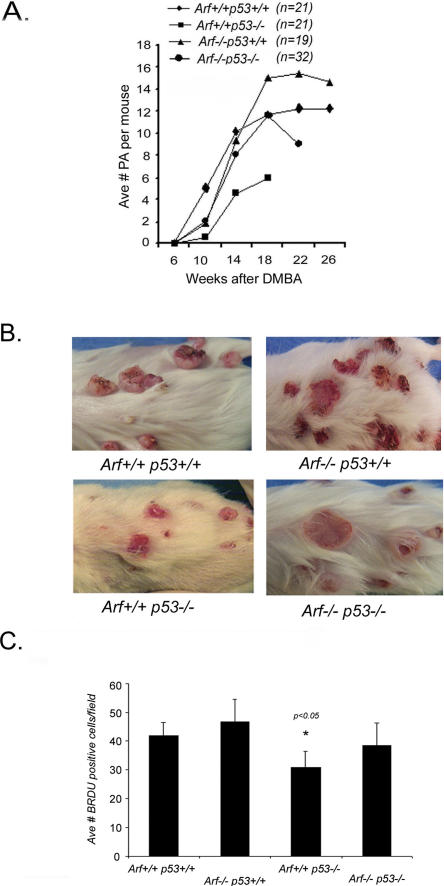
Tumor Multiplicity and Proliferative Index in *p19^ Arf^ /p53* Compound Mutant Mice (A) Average number of papillomas (more than 2 mm in diameter) per mouse is plotted against the number of weeks post-initiation. (B) Image of wild-type, *p19^ Arf^ (Arf)^−/−^, p53^−/−^,* and *p19^Arf−/−^p53^−/−^* mice with skin tumors at time of sacrifice. Wild-type mice show large exophytic tumors, while both *p19^ Arf^*- and p53-deficient mice have endophytic tumors. Note larger tumors in *p19^Arf^ /p53* compound mutant mice relative to *p53* single mutants. (C) BrdU-positive cells in papillomas from wild-type*, p53^−/−^, p19^ Arf−/−^,* and *p19^ Arf−/−^p53^−/−^* mice at 10 wk postinitiation. (Bars represent average counts ± standard deviation from ten fields and five mice). *p53^−/−^* tumors show significantly fewer BrdU-positive cells than either *p19^ Arf−/−^* or wild-type tumors (*p* < 0.05, Wilcoxon one-sided t-test).

The observations that p19^Arf^ and p53 were upregulated in papillomas, that p53 expression was reduced in *p19^ Ar^* ^f^-null papillomas, and that loss of p19^Arf^ had a similar effect on tumor progression as that of loss of p53, provide strong in vivo support of the model whereby p19^Arf^ regulates p53 in response to mutational activation of *Hras*. However, enhanced tumor growth in *p19^ Arf^*-null mice, in contrast to reduced tumor growth in *p53*-null mice (Kemp et al. 1993), suggests additional tumor suppressor functions of p19^Arf^, independent of p53. To examine the effect of the combined loss of p53 and p19^Arf^ tumor suppressors, skin tumors were induced in *p19^ Arf^* and *p53* single and compound mutant littermates. Relative to wild-type mice, *p53*-null mice developed fewer tumors, averaging 4.05 fewer papillomas (95% CI (−6.10, −2.00); *p* = 0.0001) 10–16 wk after the DMBA administration, while *p19^ Arf^*-null mice averaged 2.68 more papillomas (95% CI (0.52, 4.84); *p* = 0.015) 18–40 wk after the DMBA administration (Figure 5A). The *p19^Arf^ /p53* double-null mice showed a papilloma multiplicity similar to wild-type mice. *p53* ^−/−^ tumors were also smaller, while both *p19^Arf−/−^* and *p19^Arf−/−^p53^−/−^* tumors were larger compared to wild-type tumors. p19^Arf^ and p53 also affected tumor size and morphology. Wild-type papillomas were highly exophytic, while tumors from both *p19^ Arf^-* and *p53-*deficient mice grew in a flatter, endophytic pattern (Figure 5B). Thus, loss of p19^Arf^ increased the number and size of both wild-type *p53* and *p53*-null tumors, demonstrating that p53 and p19^Arf^ contribute independently to the early stages of tumor development.

To determine whether tumor growth in mice lacking p19^Arf^ or p53 is due to altered proliferation, additional cohorts of mice were treated with DMBA/TPA. Tumor-bearing mice 8–10 wk post DMBA treatment were injected with BrdU and sacrificed 1 h later. *p53*-null papillomas showed a reduced BrdU labeling index compared to wild-type mice, while the *p19^ Arf^*-null tumors showed a higher BrdU labeling index (Figure 5C). The *p19^ Arf^ /p53* double-null papillomas showed a proliferative index similar to that of wild-type tumors. Thus, decreased proliferation contributes to the reduced tumor growth seen in the *p53*-null mice. Apoptotic cells were very rare in papillomas regardless of *p53* genotype or radiation exposure (apoptotic index over 40-fold less than proliferation index) (unpublished data). Thus, p53-regulated apoptosis does not appear to play a major role in SCC development, at least at the papilloma stage.

### Tumor Progression in *p19^Arf^ /p53* Compound Mutant Mice

**Figure 6:**
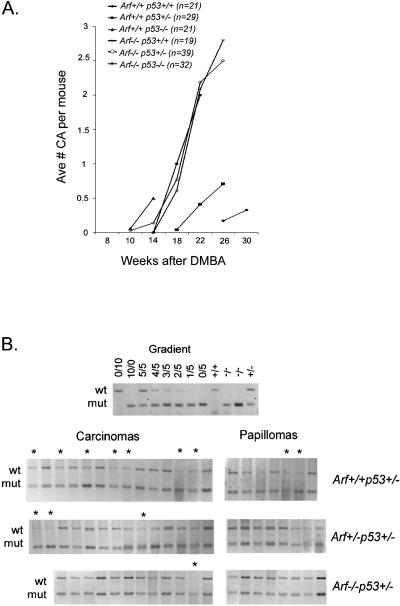
Tumor Progression and *p53* LOH in *p19^Arf^ /p53* Compound Mutant Mice (A) Average number of carcinomas per mouse is plotted against the number of weeks postinitiation. Tumor progression was accelerated in all *p19^ Arf^ (Arf)-* and *p53*-deficient genotypes compared to wild-type littermates*.* Carcinoma latency and multiplicity was almost identical for *p19^ Arf−/−^* mice regardless of *p53* genotype (*p53^+/+^, p53^+/−^,* or *p53^−/−^*). (B) LOH of the wild-type *p53* allele by semiquantitative PCR in *p19^ Arf^ /p53* compound tumors. Gradient made from kidney DNA used for quantitation of wt/mu ratio (top row). wt, wild-type allele; mu, knockout allele; asterisk, tumors with reduction of wild-type *p53.*

To determine if p19^Arf^ and p53 cooperate during tumor progression, papilloma to carcinoma conversion was evaluated in *p19^Arf−/−^, p53^−/−^,* and *p19^Arf−/−^p53^−/−^* littermates. Tumor progression was accelerated in all *p19^ Arf^-* and *p53*-deficient genotypes compared to wild-type littermates (Figure 6A). Carcinoma latency and multiplicity was almost identical for *p19^ Arf−/−^* mice regardless of *p53* genotype *(p53^+/+^, p53^+/−^,* or *p53^−/−^)* (Figure 6A), indicating no cooperation between p19^Arf^ and p53 for malignant conversion per se. However, the size of carcinomas in both *p19^ Arf−/−^* and *p19^ Arf−/−^p53^−/−^* mice was considerably greater than that seen in *p53^−/−^* mice at comparable time points (see Figure 5B). This confirms a significant impact of p19^Arf^ on suppressing tumor growth that does not require p53.

In *p53^+/−^* mice there is strong selective pressure to lose the wild-type allele during conversion to malignancy (Kemp et al. 1993). As p19^Arf^ regulates p53, we next wished to determine if the selective pressure to lose p53 was reduced in the absence of p19^Arf^. Tumors from *p53^+/−^* mice of all three *p19^ Arf^* genotypes *( p19^ Arf+/+^, p19^ Arf+/−^,* and *p19^ Arf−/−^)* were assessed for LOH of *p53* by semiquantitative PCR analysis of genomic DNA. Two out of seven papillomas from *p19^ Arf+/+^p53^+/−^* mice show loss of the remaining *p53* allele, while all papillomas examined from *p19^ Arf+/−^p53^+/−^* (*n* = 8) and *p19^ Arf−/−^p53^+/−^* (*n* = 8) mice show retention of wild-type *p53* (Figure 6B). Seven of 14 (50%) carcinomas from *p19^ Arf+/+^p53^+/−^,* and three of 14 (21%) from *p19 ^Arf+/−^p53^+/−^,* but only one of 12 (8%) from *p19^ Arf−/−^p53^+/−^* (*p* = 0.036) showed loss of *p53*. Thus, deletion of p19^Arf^ disrupts the activation of p53 and thereby reduces selection for mutations in *p53* during malignant progression.

## Discussion

Using a multistage model of tumor progression, we have examined the functional interactions between the oncogene *Hras* and the tumor suppressors *p19^Arf^* and *p53*. Somatic mutation of *Ras* is an early and frequent event in this model of tumor development. Against this backdrop, p19^Arf^ has at least two distinct tumor suppressor properties, which act at different stages of tumor development and which show a range of gene dosage effects. Loss of one or both *p19^ Arf^* alleles leads to accelerated growth of benign tumors, indicating *p19^ Arf^* is partially haploinsufficient for suppression of this early growth phenotype. Although p19^Arf^ regulates p53 at this stage, suppression of tumor growth per se by p19^Arf^ does not appear to be mediated through p53. p19^Arf^ also inhibits the benign to malignant transition and subsequent tumor cell dissemination and metastasis, and this effect of p19^Arf^ is, in contrast, mediated through p53. LOH of *p19 ^Arf^* occurs preferentially in malignant tumors, indicating complete loss of p19^Arf^ is favored during progression. Thus, p19^Arf^ inhibits several stages in Ras-driven tumor progression. Furthermore, Ras is connected to both p19^Arf^ and p53 through a signaling pathway, indicating that selection for mutations in the p19^Arf^ / p53 pathway are a direct consequence of the initial *Ras* mutation.

### Ras, p19^Arf^, p53, and Early Tumor Growth

The observation that *Hras* mutations are found at high frequency in papillomas from wild-type and both p19^Arf^- and p53-deficient mice indicates that squamous epithelial cells harboring *Hras* mutations have a strong selective advantage, with or without the presence of p19^Arf^ or p53. This permits analysis of the effects of Ras on defined genetic backgrounds in the natural setting of tumor cell evolution. The expression levels of both p19^Arf^ and p53 were increased in wild-type papillomas but not in *p19^ Arf−/−^* papillomas, indicating that p19^Arf^ regulates p53 in response to activated Ras in vivo. However, other signals to induce p53, such as those stemming from DNA damage, remain intact in the absence of p19^Arf^, as shown by the rapid increase in p53 in irradiated *p19^ Arf^*-null tumors. Thus, of the many stimuli that have been shown to activate p53 using a variety of experimental systems (Ko and Prives 1996; Giaccia and Kastan 1998), the Ras-p19^Arf^ pathway appears to be the major signal that operates during SCC development. This indicates that p53 activation is an intrinsic consequence of the oncogenic pathway that drives tumor growth, and is not due to other microenvironmental factors (e.g., those induced by hypoxia or due to lack of survival factors) or exogenous stimuli (e.g., DNA damage inducers). However, it remains possible that these other modes of p53 activation might predominate in other tumor types or in other circumstances.

Despite the fact that both p19^Arf^ and p53 were induced in papillomas, loss of p19^Arf^ or p53 had opposite effects on early tumor growth. p19^Arf^ deficiency resulted in increased tumor cell proliferation and tumor growth while p53-deficient mice had reduced tumor cell proliferation, tumor numbers, and tumor size. The observation that tumors harboring mutant *Ras* grew faster in the presence of p53 than in the absence of p53 differs from in vitro studies, in which ectopically expressed Ras induces p53-dependent growth arrest or senescence (Palmero et al. 1998; Zindy et al. 1998; Lin and Lowe 2001). These different outcomes are likely due to different experimental conditions. Ex vivo culture per se induces stress, which can further induce p53 and accelerate senescence (Lowe and Ruley 1993; Serrano et al. 1997; Sherr and DePinho 2000). Moreover, the levels of active Ras protein likely differ; our autochthonous tumor model begins with a single cell that has undergone a mutation at the endogenous *Hras* locus, and subsequent tumor growth occurs in the context of surrounding normal cells. Locally produced growth factors or TPA treatment may attenuate the effect of Ras on p53, effectively dampening the response. Thus, the absolute levels of both Ras and p53, as well as cell type involved and the local cellular ecology, may dictate the outcome.

Greenhalgh al. (1996) also reported reduction in *Ras*-transgene-induced skin tumors in a *p53*-null background. We suggest that the acute effect of a constitutively active oncogene driving cellular proliferation, combined with lack of cell cycle checkpoints due to p53 deficiency, may generate excessive genetic instability, which may initially impair overall cellular fitness. This may be especially true at the early post-initiation stage where a small number of incipient tumor cells are competing with surrounding normal cells. This idea is consistent with the longstanding observation that p53-deficient mice (Donehower et al. 1992), and Li-Fraumeni patients who carry a germline *p53* mutation (Vogelstein 1990), rarely develop multiple tumors, which would be expected if loss of p53 provided an early selective advantage. Also, in many human malignancies, mutations in *p53* are infrequent in early premalignant lesions and much more common in late-stage disease, indicating a long latency between oncogene activation and loss of p53. Further genetic or epigenetic changes may be required for a cell to adapt to the combined effect of a dominant oncogene and loss of p53 to gain a fitness advantage. Loss of p19^Arf^ appears to be one such change, as reduced tumor growth due to the absence of p53 was rescued by loss of p19^Arf^.

Although p19^Arf^ regulates p53, suppression of tumor growth per se by p19^Arf^ does not appear to be mediated through p53. Other reports have suggested that p19^Arf^ has tumor suppressor functions that are independent of p53. *p19^ Arf^*-null mice show a broader spectrum of spontaneous tumors compared to *p53*-null mice (Donehower et al. 1992; Kamijo et al. 1999). Mice lacking both *p19 ^Arf^* and *p53* showed a wider range of tumor types than animals lacking either gene alone, and many developed multiple primary tumors (Weber et al. 2000; Moore et al. 2003). Premalignant B-cells expressing oncogenic Eμ-myc and lacking both *p19^ Arf^* and *p53* proliferated at a faster rate than cells lacking either *p19^ Arf^* or *p53* alone (Weber et al. 2000). Indeed, both p19^Arf^ and p53 are lost in a wide spectrum of human cancers, both familial and sporadic, at very high frequency (Ruas and Peters 1998; Vonlanthen et al. 1998). Microarray and GeneChip analysis of genes induced by a conditionally regulated p19^Arf^ has identified members of the B-cell translocation gene family whose induction is independent of p53 (Kuo et al. 2003). Expression of these genes inhibits cell proliferation and induces cell cycle arrest. p19^Arf^ can colocalize with the human replication protein A, suggesting a direct role for p19^Arf^in DNA synthesis (Yarbrough et al. 2002). p19^Arf^ has also been shown to inhibit ribosomal RNA processing (Sugimoto et al. 2003) and to repress NF-κB transactivation (Rocha et al. 2003). Finally, p19^Arf^ regulates vascular regression independent of p53, suggesting a role for p19^Arf^ in angiogenesis. (McKeller et al. 2002). The functional relevance of these phenotypes for tumor suppression by p19^Arf^ remains to be elucidated.

### Ras, p19^Arf^, p53, and Malignant Progression

In the DMBA/TPA model, conversion of papillomas to carcinomas is a relatively rare event and can take up to 6–12 mo. Phenotypes associated with conversion include loss of basement membrane integrity, invasion of epithelial tumor cells into the dermis, loss of differentiation, and increased cellular atypia. Even more rarely, these carcinoma cells can metastasize, which involves additional phenotypic changes including, extravasation, migration, attachment, and establishment of tumor growth in an ectopic tissue. Although benign tumor growth differed between p19^Arf^- and p53-deficient mice, both mutant mice showed dramatically accelerated progression to malignancy and rapid metastasis. Thus, benign tumors lacking either p19^Arf^ or p53 are at high risk for metastasis. In p19^Arf^-deficient mice, progression was similar with or without the presence of p53 and did not involve *p53* LOH, indicating that loss of p19^Arf^ decreased selection for *p53* mutations during progression and that p19^Arf^ acts through p53 at this stage. Schmitt et al. (1999) also reported increased lymphoma dissemination in Eμ-myc *p53^−/−^* mice relative to Eμ-myc mice alone. From a clinical perspective, then, the most relevant effect of the p19^Arf^-p53 pathway may be to inhibit malignant conversion and metastasis. Loss of p19^Arf^ and/or p53 can increase progression and metastasis by several mechanisms. Deficiency in p19^Arf^ or p53 could indirectly affect progression via increased genetic instability, increased generation of mutants, and accelerated tumor evolution. This view postulates the existence of a distinct class of genes whose dysfunction increases progression and metastasis. It also requires a series of clonal evolutionary steps to select for cells carrying mutations in these genes. Alternatively, loss of p19^Arf^ or p53 can directly affect cellular phenotypes associated with progression through transcriptional regulation, or by relieving inhibition of Ras signaling.

Both p19^Arf^- and p53-deficient papillomas displayed several characteristics consistent with early malignancy. Conversion of papillomas to carcinomas is first characterized by a flattening, endophytic transition. p19^Arf^- and p53-deficient papillomas had this morphology from the outset, suggesting an early propensity for malignant conversion. More detailed histological and immunochemical characterization showed that p53-deficient papillomas were more dysplastic and had aberrant expression of differentiation markers such as E-cadherin, P-cadherin, and Keratin-13 (Cano et al. 1996). E-cadherin is a critical component of cell–cell adhesion and its down regulation is strongly associated with malignant progression (Birchmeier et al. 1993; Perl et al. 1998). That these “high risk” *p53*-null papillomas exhibited these early malignant features argues for a more direct effect of p53 on cellular phenotypes associated with progression. In addition, there was a lack of correlation between papilloma size and propensity to progress. *p53*-null papillomas were fewer and smaller, yet these showed the most rapid progression. Finally, metastasis of skin tumors in both p19^Arf^- and p53-deficient mice was observed within a matter of days after papilloma to carcinoma conversion, again, irrespective of precursor tumor size. Collectively, these data are inconsistent with a model in which loss of p19^Arf^ or p53 indirectly accelerates tumor progression by accelerating a series of independent genetic events, each followed by clonal selection, and instead favor a more direct model.

We suggest that Ras may be a major driving force for multiple steps in tumor progression, with loss of p19^Arf^ and p53 playing a facilitating role. In addition to the well-known effects of Ras on proliferation, consistent with an early role in neoplasia, Ras also contributes to a number of phenotypes that are involved in malignant progression, including metastasis. Ras induces cell motility, invasiveness (Lazarov et al. 2002; Dajee et al. 2003; Kim et al. 2003), epithelial to mesenchymal transition (Oft et al. 1996; Zondag et al. 2000), angiogenesis (Arbiser et al. 1997; Casanova et al. 2002), and metastasis (Pozzatti et al. 1986; Webb et al. 1998; Oft et al. 2002; Varghese et al. 2002). Ras activates a number of signaling cascades that drive these processes (Campbell et al. 1998). For example, Ras activation of the Raf-MAPK signaling cascade regulates the activities of nuclear transcription factors, including AP-1 (Campbell et al. 1998). In addition to regulating proliferation, AP-1 induces a motility/invasion program (Ozanne et al. 2000; Jochum et al. 2001; Young et al. 2003). Ras transgenic mice that lack Fos, a component of AP-1, develop benign skin tumors, but these fail to convert to carcinomas (Saez et al. 1995). MAPK activation also determines the ability of Ras-transformed fibroblasts to metastasize to the lung (Webb et al. 1998). Oncogenic Ras also works in concert with the Rho family of GTPases to regulate the intracellular actin cytoskeleton and promote cell motility and invasion leading to metastasis (Zohn et al. 1998). Sustained signaling by oncogenic Ras can permanently downregulate Rac activity and lead to an epithelial to mesenchymal transition. This transition is associated with changes in gene expression, loss of E-cadherin-mediated cell–cell adhesions, and increased invasiveness of tumor cells (Oft et al. 1996, 2002; Zondag et al. 2000). This allows the cell to become mobile, invade surrounding tissue, and establish metastatic sites. An additional link between Ras and tumor progression was demonstrated in a genome-wide survey of Ras transformation targets that identified a significant increase in expression of genes triggering invasion and metastasis, such as the laminin receptor, collagenase (Mmp-1), stromolysin (Mmp-3), and the Cd44 glycoprotein (Zuber et al. 2000). Ras also repressed genes involved in anti-invasive or anti-angiogenic activity, such as syndecan-2, tissue inhibitor of metalloproteases-2 (Timp-2), and thrombospondin-1.

Further support for a continual involvement of Ras in tumor progression is indicated by the increase in copy number of mutant *Ras* alleles that is observed during tumor progression. In the DMBA/TPA skin tumor model, the mutant *Ras* allele is frequently duplicated in papillomas and amplified to multiple copies in carcinomas, especially in the most aggressive spindle cell tumors (Bremner and Balmain 1990; Buchmann et al. 1991). Increased expression of mutant *Ras* genes by gene amplification or other mechanisms is found in other tumor types (Schwab et al. 1983; Tanaka et al. 1986; Winter and Perucho 1986; Yokota et al. 1986) and has been shown to promote the growth of head and neck SCC and carcinoma of the cervix (Hoa et al. 2002; Soh et al. 2002). This indicates that there is a selective advantage to progressively increasing levels of Ras throughout tumor progression. Quantitative differences in Ras activity are known to differentially activate signaling pathways leading to different cellular outcomes (Shields et al. 2000). Loss of p53 may facilitate the increase in Ras levels, in that cells with inactivated p53 show greatly increased frequency of gene amplification (Yin et al. 1992).

**Figure 7:**
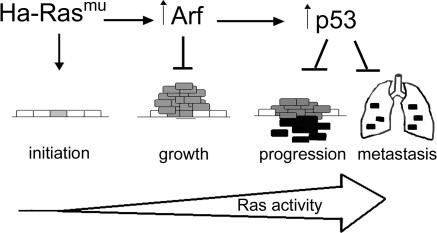
An Integrated Model of SCC Progression At the genetic level, treatment of mouse skin with DMBA induces mutations in *Hras* resulting in initiated cells that express constitutively active Ras protein (grey rectangles). TPA treatment is required for clonal expansion of these *Hras* mutant cells to form papillomas. Frequent duplication of the mutant *Ras* allele in papillomas indicates increased Ras signaling is favored. Mutation of *p53,* as well as additional *Ras* gene amplification, is seen in carcinomas, particularly in the most aggressive tumors (black rectangles). At the signaling level, mutant Ras upregulates p19^Arf^ (Arf), which in turn activates p53. p19^Arf^, in turn, inhibits growth of Hras-driven tumors in a p53-independent manner. p19^Arf^, acting through p53, also inhibits malignant progression and metastasis. As Ras signals through p19^Arf^ and p53, selection for subsequent mutations in *p19^ Arf^* or *p53* is a direct consequence of the initial *Ras* mutation. In this model, Ras drives tumor progression through direct signaling effects and by dictating the evolution pathway of the tumor.

Thus, mutation in *Ras* is much more than the initiating event: it directly contributes to many of the phenotypes associated with malignant progression (Figure 7). As Ras induces both p19^Arf^ and p53, and both are antagonistic to Ras, we suggest that an important consequence of p19^Arf^ and p53 loss is that it permits increased Ras levels and signaling, fueling further tumor progression. In addition to counteracting Ras, p19^Arf^ and p53 likely contribute to tumor suppression through additional pathways. For example, loss of p19^Arf^ increases tumor growth, and loss of p53 confers resistance to apoptosis and loss of cell cycle checkpoints, leading to genetic instability. The observation that oncogene mutations are linked to tumor suppressor gene activation through mechanistic signaling pathways indicates that selective pressure in favor of tumor suppressor gene mutations originates from the initial oncogenic lesion and is thus intrinsic to the tumor.

## Materials and Methods

### 

#### Mice

p19^Arf^-deficient mice (C57BL/6 × 129SvJ) were provided by Martine Roussel and Charles Sherr (Kamijo et al. 1997). To increase their susceptibility to skin tumor development, *p19^ Arf−/−^* mice were backcrossed to the susceptible NIH/Ola strain (Harlan Olac, Oxfordshire, United Kingdom), and carcinogenesis studies were performed on the F_3_ littermates of this cross. 20 mice of each genotype, *p19^ Arf−/−^, p19^ Arf+/−^,* and *p19^ Arf+/+^* were treated. The backs of 8-wk-old male and female mice were shaved and treated with a single application of DMBA (25 μg in 200 μl of acetone; Sigma, St. Louis, Missouri, United States) followed a week later by twice weekly applications of TPA (200 μl of 10^−4^ M solution in acetone) for 15 wk. The number and size of papillomas on each mouse were recorded every 2 wk. Mice were sacrificed if moribund or following detection of carcinomas. Tumors were frozen for DNA extraction and/or fixed in formalin to be processed and stained with hematoxylin/eosin (H&E) for histological examination.

Mice deficient for *p53* (Donehower et al. 1992) (F_7_ backcross to NIH) were crossed to *p19^ Arf^*-deficient (F_4_ backcross to NIH) mice to generate *p19^  Arf+/−^ p53^+/−^* mice. These mice were intercrossed to generate all nine possible *p19^ Arf^ /p53* genotypes*.* Some 20–30 mice of each genotype were subjected to the same DMBA/TPA protocol and monitored as described above.

#### Immunoblotting

In order to remove abundant keratin present in papillomas and carcinomas, nuclear extracts were prepared as described (Schreiber et al. 1989) with modifications. Pieces of skin or tumor were ground in liquid nitrogen with a mortar and pestle, and the resulting powder was dissolved in buffer A and further homogenized for 1 min on ice (PowerGen 125, Fischer Scientific, Pittsburgh, Pennsylvania, United States). Buffers A and C both contained 1 mM DTT, 0.4 mg/ml Pefablock, 25 mg/ml Aprotinin, 10 mg/ml Pepstatin, and 10 mg/ml leupeptin (Roche, Alameda, California, Unites States) to inhibit proteases. Protein concentrations were standardized using the Bradford assay (Bio-Rad, Hercules, California, United States) and equal loading (50 μg/lane) was confirmed by Ponceau S staining of the nylon membrane after blotting. Western blot analysis was performed using specific antibodies against p19^Arf^ (Novus Biologicals, Littleton, Colorado, United States) and p53 (Novocastra Laboratories, Newcastle-upon-Tyne, United Kingdom).

#### *Hras* sequencing

Genomic DNA was prepared from tumor and normal tissue by QIAamp DNA Mini Kit (Qiagen, Valencia, California, United States). A 400-bp PCR fragment containing exon 2 of *Hras* was amplified with standard PCR (3.0 mM MgCl_2_ andannealing for 2 min at 37 °C with 40 cycles), with 5′-GACTCCTACCGGAAACAGGT-3′ and 5′-CTGTACTGATGGATGTCCTC-3′ primers. We used the internal primer 5′-TGGTCATTGATGGGGAGACA-3′ to sequence exon 2, using PE Biosystems (Applied Biosystems, Foster City, Calfornia, United States) Dye-Terminator and Big-Dye cycle sequencing.

#### Histological analysis

Sections of normal skin, papillomas, carcinomas, and other organs were removed and fixed in 10% normal buffered formalin for 4 h. After fixation, tissues were processed and then embedded in paraffin. From the tissues, 4-μm sections were cut and stained for either p53 (CM5, Novocastra) or pan-keratin (AE1/AE3, Novocastra) using high-temperature antigen retrieval in 10 mM citrate buffer (pH 6), or for BrdU (Dako, Glostrup, Denmark) after treating with 2N HCl followed by 0.1% trypsin. After staining with the primary antibody, the sections were stained with a biotin-conjugated secondary (Vector Laboratories, Burlingame, California, United States) followed by StreptABComplex/HRP (Dako). Slides were developed with DAB/NiCl and counterstained with methyl green. Control sections with no primary antibody were run concurrently. Other sections were cut and stained using a standard H&E method. Proliferation index was determined by counting the number of BrdU-stained cells per 40× field. The apoptotic index was determined by counting the H&E slides for the number of apoptotic figures per 40× field. All counts were done on a Nikon (Tokyo, Japan) Labophot-2 microscope.

#### Semiquantitative PCR analysis

Genomic DNA was prepared from tumor tissue or normal kidney by QIAamp DNA Mini Kit (Qiagen). To measure LOH of the *p19^Arf^* wild-type allele in *p19^Arf+^* ^/−^ tumors, wild-type and knockout alleles were amplified by PCR separately then combined for electrophoresis. Primers 5′-AGTACAGCAGCGGGAGCATGG-3′ *(Arf1),* 5′-TTGAGGAGGACCGTGAAGCCG-3′ *(Arf2),* and 5′-ACCACACTGCTCGACATTGGG-3′ *(ArfN)* were used to amplify wild-type *(Arf1* and *Arf2)* and knockout alleles *(Arf2* and *ArfN)* from 100 ng of genomic DNA using 68 °C for annealing and extension at 90 s for 30 cycles. Equal amounts of each PCR product were then combined for electrophoresis on a 2% TAE agarose gel. Wild-type and knockout alleles of *p53* ^+/−^ tumors were amplified in a separate reaction as described (Timme and Thompson 1994) for 30 cycles. PCR products were electrophoresed on a 2% TAE agarose gel. Comparison gradients for *p19^Arf^* and *p53* were established by combining wild-type and knockout genomic DNA in quantified ratios, then amplifying as described above.

#### Statistical methods

In order to assess the impact of *p19^Arf^* (or *p19^Arf^ /p53*) genotypes on the development of papillomas, longitudinal profiles of papilloma counts were analyzed using the generalized estimating equation (GEE) approach (Zeger and Liang 1986). GEE is an established statistical approach to the regression analysis of longitudinal data. Our analysis used papilloma counts of each mouse measured every 2 wk as the outcome variable and incorporated within-mouse correlations of papilloma counts over time in making statistical inference. Using GEE, we estimated average differences of papilloma counts across genotypes after DMBA administration. Since the development of papillomas depends on the time since the DMBA administration and may differ by the sex of the mice, the effects of the weeks since the DMBA administration and sex were controlled for in the GEE analysis as covariates. A working correlation structure of the GEE analysis was specified as the first-order autoregressive structure over the time since the DMBA administration; however, GEE is robust against the misspecification of the correlation structure. In contrast to papillomas, the overall number of carcinomas developed during the experiment was relatively small. Thus, we analyzed differences by genotype in the probability of developing a carcinoma after DMBA administration. A logistic regression model (Clayton and Hills 2003) was used to assess the odds of developing a carcinoma during the experimental period and to compare it across *p19^Arf^* (or *p19^Arf^ /p53*) genotypes. Estimates of relative odds were adjusted for sex effects. Fisher's exact test was used for comparing two proportions such as comparing LOH proportions between papillomas and carcinomas. All statistical tests were two-sided.

## Abbreviations

Arf: p19^Arf^
GEE: generalized estimating equation
H&E: hematoxylin/eosin
LOH: loss of heterozygosity
SCC: squamous cell carcinoma
